# Assessment of Suitable Reference Genes for Quantitative Gene Expression Studies in Melon Fruits

**DOI:** 10.3389/fpls.2016.01178

**Published:** 2016-08-03

**Authors:** Qiusheng Kong, Lingyun Gao, Lei Cao, Yue Liu, Hameed Saba, Yuan Huang, Zhilong Bie

**Affiliations:** Key Laboratory of Horticultural Plant Biology, Ministry of Education; College of Horticulture and Forestry Sciences, Huazhong Agricultural UniversityWuhan, China

**Keywords:** *Cucumis melo*, fruit development, gene expression, reference gene, normalization, qRT-PCR

## Abstract

Melon (*Cucumis melo* L.) is an attractive model plant for investigating fruit development because of its morphological, physiological, and biochemical diversity. Quantification of gene expression by quantitative reverse transcription polymerase chain reaction (qRT-PCR) with stably expressed reference genes for normalization can effectively elucidate the biological functions of genes that regulate fruit development. However, the reference genes for data normalization in melon fruits have not yet been systematically validated. This study aims to assess the suitability of 20 genes for their potential use as reference genes in melon fruits. Expression variations of these genes were measured in 24 samples that represented different developmental stages of fertilized and parthenocarpic melon fruits by qRT-PCR analysis. GeNorm identified *ribosomal protein L* (*CmRPL*) and *cytosolic ribosomal protein S15* (*CmRPS15*) as the best pair of reference genes, and as many as five genes including *CmRPL, CmRPS15, TIP41-like family protein* (*CmTIP41*), *cyclophilin ROC7* (*CmCYP7*), and *ADP ribosylation factor 1* (*CmADP*) were required for more reliable normalization. NormFinder ranked *CmRPS15* as the best single reference gene, and *RAN GTPase gene family* (*CmRAN*) and *TATA-box binding protein* (*CmTBP2*) as the best combination of reference genes in melon fruits. Their effectiveness was further validated by parallel analyses on the activities of soluble acid invertase and sucrose phosphate synthase, and expression profiles of their respective encoding genes *CmAIN2* and *CmSPS1*, as well as sucrose contents during melon fruit ripening. The validated reference genes will help to improve the accuracy of gene expression studies in melon fruits.

## Introduction

Melon (*Cucumis melo* L.) is a cucurbitaceous crop cultivated worldwide, and it is one of the most important fleshy fruits used for fresh consumption. Melon fruits exhibit extreme diversity in terms of shape, size, flesh, color, sweetness, aroma, and fruit texture (Nunez-Palenius et al., 2008). In addition, melon fruits have significant variations in their ripening physiology; the fruits can be categorized as either climacteric or non-climacteric types based on their ripening-related respiration rate and ethylene evolution profiles (Clepet et al., 2011; Leida et al., 2015; Saladié et al., 2015). These diverse traits can be exploited to reveal the underlying biological processes and mechanisms regulating fruit development. Accordingly, melon is considered an alternative model plant for elucidating fruit ripening (Ezura and Owino, 2008). Extensive molecular and genetic studies have been conducted on melon in recent years to understand the regulatory mechanisms of fruit development and ripening, with the aim of improving its fruit quality (Moreno et al., 2008; Dai et al., 2011; Portnoy et al., 2011; Vegas et al., 2013; Díaz et al., 2014; Saladié et al., 2015).

Gene expression data during melon fruit development is crucial to study the fruit expansion and maturity mechanisms. Quantitative reverse transcription polymerase chain reaction (qRT-PCR) has currently become the widely used method for quantification of target gene expression because of its sensitivity and rapidness. However, qRT-PCR is a multiple-step method and its use is inherently variable, which may cause the gene expression data to differ from the actual data (Nolan et al., 2006). To overcome this limitation of the technique and to ensure its accurate results, a robust normalization strategy is applied by using reference genes that have been shown to be stably expressed under the experimental conditions (Dheda et al., 2005; Huggett et al., 2005). Consequently, selection of reference genes with stable expression is a very important step prior to qRT-PCR analysis (Gutierrez et al., 2008).

The quantification of gene expressions in melon fruits by qRT-PCR have been conducted in several studies, and different reference genes were used for normalization, such as *actin* (*ACT*; Shan et al., 2012; Sun et al., 2013), *cyclophilin* (*CYP*; Gonzalez-Ibeas et al., 2007; Portnoy et al., 2011; Qin et al., 2011; Galpaz et al., 2013; Gonda et al., 2013), and *glyceraldehyde-3-phosphate dehydrogenase* (*GAPDH*; Gao et al., 2013). However, the expression stability of these genes in melon fruits has not been systematically assessed to date. Validations of reference genes have been performed in roots, leaves, and stems of melons (Kong et al., 2014b; Sestili et al., 2014), but not in fruits and other organs. Reference genes have been proven to be organ-specific or tissue-specific in several studies (Guénin et al., 2009; Kong et al., 2015). Therefore, the reference genes validated in roots, stems, and leaves of melons may be inappropriate for normalizing the target genes in melon fruits. The lack of validated reference genes specifically for melon fruits will certainly affect the accurate quantification of gene expression in melon fruits. Meanwhile, the availability of genome sequence (Garcia-Mas et al., 2012) and large-scale transcriptome data (Clepet et al., 2011; Portnoy et al., 2011; Saladié et al., 2015) will inevitably expedite the functional genomics research on melons, particularly on melon fruits. Therefore, selection of organ-specific reference genes with stable expression in melon fruits is vital to accurately explain the gene expression profiles during melon fruit development.

Fertilization and parthenocarpy are fruit set methods widely used for commercial melon production (Shin et al., 2007). In open field, fertilized fruits are often produced through natural pollination, while, under greenhouse conditions, parthenocarpic fruits are induced by the exogenous application of *N*-(2-chloro-4-pyridyl)-*N*′-phenylurea (CPPU). CPPU is a synthetic cytokinin and can induce parthenocarpic fruit development in the absence of pollination and fertilization (Nagasawa et al., 2005). The two fruit set methods will trigger different patterns of gene expression and regulatory mechanisms during the subsequent fruit development, thereby providing a good opportunity for the selection of reference genes with intrinsically stable expression during melon fruit development. The sugar content and composition are major criteria used for assessment of melon fruit quality. Sucrose is the predominant sugar found in ripe melon fruits. Some previous studies have shown that soluble acid invertase (AI) and sucrose phosphate synthase (SPS) are the primary determinants closely associated with sucrose accumulation in melon fruits (Hubbard et al., 1989; Burger and Schaffer, 2007). Two AI genes (*CmAIN1* and *CmAIN2*) and two SPS genes (*CmSPS1* and *CmSPS2*) have been identified in the melon genome (Dai et al., 2011; Garcia-Mas et al., 2012). However, only *CmAIN2* and *CmSPS1* have been associated with sucrose accumulation in ripening melon fruits by deep sequencing analysis (Dai et al., 2011).

In this study, 20 candidate reference genes were selected, and their expression stability was evaluated at different development stages of fertilized and parthenocarpic melon fruits, with the aim of determining optimal reference genes for accurate quantification of target genes in melon fruits. Moreover, parallel analyses on the expression profiles of *CmAIN2* and *CmSPS1* normalized by the identified reference genes, and enzyme activities of AI and SPS, as well as sucrose accumulation during melon fruit ripening were performed to demonstrate the reliability of the identified reference genes.

## Materials and Methods

### Plant Materials and Treatments

Melon (*Cucumis melo* L. var. *inodorus* cv. ‘*Elizabeth*’) plants were grown in a plastic greenhouse under commercial production conditions at the Huazhong Agricultural University (East Longitude 113°41′ 115°05′, North Latitude 29°58′ 31°22′). This cultivar is monoecious and without hermaphroditic flowers. To get fruits, two different fruit set methods were used, i.e., CPPU treatment and artificial pollination. For CPPU treatment, female flowers were covered with paper bags 1 day before anthesis to prevent natural pollination. These paper bags were removed from the female flowers on the day of anthesis, and the unpollinated ovaries were sprayed with CPPU (Shiteyou, China) at a concentration of 10 μM in the morning. The female flowers were subsequently covered again after spraying till the onset of fruit development. Simultaneously, on other female flowers, artificial pollination was done by hand. Three biological replicates were adopted for each treatment. Fruits were harvested at 1, 3, 5, 7, 10, 15, 20, 25, 28, 30, 32, and 34 days after anthesis (DAA). Two fruits were randomly harvested at each sampling time from each biological replication and mixed together. Ovaries were sampled at 1 and 3 DAA. The mesocarp tissues in the center-equatorial portion of fruits were collected as samples after 3 DAA. All the samples were immediately frozen in liquid nitrogen and stored at -80°C for the subsequent RNA extraction, enzyme assay, and sucrose measurement.

### cDNA and DNA Preparation

The qRT-PCR protocol (Nolan et al., 2006) and 11 golden rules (Udvardi et al., 2008) were used as guidelines in the experiments. Total RNA was extracted from the ovaries or mesocarp tissues with the TransZol (TransGen, China) according to its instruction. RNA quality and quantity were measured by a NanoDrop 2000 spectrophotometer (Thermo, China), and RNA integrity was confirmed in a 2% agarose-gel electrophoresis. A PrimeScript RT Reagent Kit with gDNA Eraser (Perfect Real Time; TaKaRa, China) was used to eliminate the genomic DNA (gDNA) in the RNA samples and synthesize the cDNA. gDNA was extracted from the mesocarp tissues with a Plant Genomic DNA Kit (Tiangen, China) and amplified using 2× PCR Reagent (Tiangen, China).

### Reference Genes Selection and Primer Design

Fourteen genes were initially selected as the candidate reference genes. **Table 1** lists the information on these candidate reference genes. For each gene, BLASTN was performed via the Melonomics database^1^ against the Melon transcripts CM_3.5. The *Arabidopsis* homologs were used as the query sequences. The sequence that best matched each *Arabidopsis* query was downloaded with its respective structure information. Primers that covered exon–exon junction or flanked an intron were designed using Primer3Plus^2^. The product size was set as 80–150 bp. The specificity of the designed primers were manually verified and confirmed by running BLASTN against the Melon transcripts CM_3.5. The 2% agarose-gel electrophoresis was further used to determine the PCR amplification specificity for each gene, with gDNA and cDNA as templets. The melon species name abbreviation “*Cm*” was adopted as a prefix to specify the orthologous melon genes. To make the results comparable, six best reference genes previously reported in melon, including *ribosomal protein L2* (*L2*), *actin*^∗^, *CYP, ADP-ribosylation factor 1* (*ADP*), *ribosomal protein L* (*RPL*), and *ubiquitin extension protein* (*UBI*) that identified in stems, roots and leaves (Kong et al., 2014b; Sestili et al., 2014), were also selected. Their primer sequences were also used in the present study. The unigene accession numbers of *actin*^∗^ and *CYP* provided in the reference (Sestili et al., 2014) were used to retrieve the Melonomics database. The results showed that the two genes were annotated as *actin7* and *CYP2* on melon genome, respectively. Their annotations and gene IDs on genome were used to replace the gene names and unigene accession numbers supplied in the previous reference (Sestili et al., 2014). The prefix of “*Cm*” was also added before names of the six reported reference genes.

**Table 1:** Information on the candidate reference genes, *CmAIN2*, and *CmSPS1*.

Gene name	Description	Gene ID	Forward primer sequence (5′–3′)	Reserve primer sequence (5′–3′)	Product size (bp)	E (%)
*CmACT*	β-Actin	MELO3C023264	GAGCATCTAAACGGAGAGTTGG	GCCATCGTTTATAGATACTTGAGGA	104	98.9
*CmCAC*	Clathrin adaptor complexs mediun subunit family protein	MELO3C003397	CCATTCTCATCCAAGCCTTC	TCAACAATATCCAAAAAGACCTCA	125	100.6
*CmCYP7*	Cyclophilin ROC7	MELO3C025848	TTTACCCTCGGCGATGGAAG	TGTGAACCATTGGTGTCTGGA	134	99.4
*CmEF1α*	Elongation factor 1-α	MELO3C020441	CTGCTTGCTCCTGCGTTAAA	CCACGATGTTGATATGAGTCTTTTC	113	93.1
*CmGAPDH*	Glyceraldehyde-3-phosphate dehydrogenase	MELO3C019633	CATGGTGTTTTCAACGGAACCA	CCCATGGGATATCTGCAGGG	110	103.8
*CmPP2A*	Protein phosphatase 2A regulatory subunit A	MELO3C026508	GGCAGATAACTCAAGTTTATGGA	GCTGTAAGAGGTAAATAATCA AAGAGG	109	94.8
*CmRAN*	RAN GTPase	MELO3C026633	AAGACATCTCACAGGGGAGTT	AGCAGTGTCCCAGCAGTAAA	118	96.6
*CmRP2*	RNA polymerases II	MELO3C008589	GCGCTGGATACCAAAGGAAT	TGCGTGATCTTTACCAATGC	101	105.5
*CmRPS15*	Cytosolic ribosomal protein S15	MELO3C006471	GAAGCTGCGTAAAGCGAAAC	GGTCTTTCCATTGTAAACTCCAA	132	108.8
*CmSAND*	SAND family protein	MELO3C004874	TATCGTGGAGGAAAAGGAAGAAGC	CTCGTCCCCGTACCTGGAAT	80	105.1
*CmTBP2*	TATA-box binding protein	MELO3C015563	GGAAACATATACGGCTTTTGAGA	TTCGAAACCAAAAATCATTGC	81	107.8
*CmTIP41*	TIP41-like family protein	MELO3C018500	GGTAATCTTGTATGAGGATGAGCTG	CATCAACTCTAAGCCAGAAACG	118	107.4
*CmTUA5*	Tubulin alpha-5	MELO3C026613	AGGACTGGGACATACCGACA	CGGCTAATTTTCGCACTCGG	145	99.1
*CmYLS8*	Yellow-leaf-specific gene 8	MELO3C020882	GTGGTCATTCGTTTTGGTCA	CAGCAAAGTTCTTAATCGTCTCT	94	108.0
*CmACT7*	Actin^∗^	MELO3C008032	CCCTGGTATTGCAGACAGGA	ACATCTGCTGGAAGGTGCTT	149	105.2
*CmCYP2*	Cyclophilin	MELO3C013375	CACACCGGACCTGGTATTCT	CATCCATACCCTCGACGACT	139	107.4
*CmL2*	Ribosomal protein L2	MELO3C000111	AAACTTCTACCCCGAGCACA	TATGACCTCCCCCTCTATGC	150	109.3
*CmADP*	ADP ribosylation factor 1	MELO3C023630	ATATTGCCAACAAGGCGTAGA	TGCCCGTAAACAAGGGATAAA	93	98.2
*CmRPL*	Ribosomal protein L	MELO3C023039	CGACAATACTGGAGCCAAGAA	CATCACCATATCTCCCACACAA	100	104.6
*CmUBI*	Ubiquitin extension protein	MELO3C016083	AAGTGTGGACACAGCAACCA	AAGCCAAATGGCTCTAAGCA	132	94.5
*CmAIN2*	Acid invertase 2	MELO3C005363	AATGACGTGCTCCTCGTACC	TTCCACTTCAAACTCCGCCA	90	
*CmSPS1*	Sucrose phosphate synthase 1	MELO3C010300	GACACTTCAGTCCCACTCGG	TCTAGTATTCCTCTCCTGCGGA	122	

### qRT-PCR Analysis

Quantitative reverse transcription PCR reactions were performed on a QuantStudio 7 Flex Real-Time PCR System (The Applied Biosystems, America) using a total volume of 10 μL, which contained 0.2 μM of each primer, 1× Top Green qPCR SuperMix (TransGen, China), and100 ng of cDNA. The amplification conditions were 30 s at 94°C, 40 cycles of 5 s at 95°C, 15 s at 58°C and 10 s at 72°C, followed by a melting curve analysis by heating the PCR products from 65 to 95°C. qRT-PCR reactions were performed in two technical replicates with a negative control without template. The PCR amplification efficiency for each gene was determined by analyzing fivefold serial dilutions of pooled cDNA in the concentrations of 800, 160, 32, 6.4, and 1.28 ng μL^-1^.

### Expression Stability Analysis

The cycle threshold (Ct) value was recorded for each qRT-PCR reaction. The R statistical package^3^ was used to draw the boxplot to display the expression variation for each gene. The amplification efficiency (E) was calculated using the equation:

(1) $$E(\%)\;=\;(10^{-1/slope}-1)\;\times \;100$$

for each gene, in which the slope is the standard curve slope generated by the QuantStudio 7 Flex Real-Time PCR System based on the fivefold serial dilutions of pooled cDNA samples. The algorithms of geNorm (Vandesompele et al., 2002) and NormFinder (Andersen et al., 2004) were used to evaluate the expression variation. Prior to data entry, the raw Ct values were corrected by PCR efficiency and transformed into relative expression quantities using the equation (1 + E)^ΔCt^, in which ΔCt is the difference of the lowest Ct value of the calibrator and the Ct value of the sample being tested.

### Determination of Sucrose Contents, Enzyme Activities of AI and SPS, and Expression Patterns of *CmAIN2* and *CmSPS1* during Fruit Ripening

Pollinated fruits at 10, 15, 20, 25, 28, and 32 DAA were used to determine the sucrose contents and enzyme activities. Extraction and measurement of sucrose were performed as previously described (Liu et al., 2012). The gas chromatograph Agilent 7890A (Agilent Technologies, USA) coupled with a HP-5 capillary column (30 m × 0.32 mm × 0.25 μm) and a CTC PAL autosampler (CTC Analytics, Switzerland) were used for sucrose detection and quantification. Extractions and activity measurements of AI and SPS were conducted as previously reported (Hubbard et al., 1989). *CmAIN2* and *CmSPS1* were selected to test the effectiveness of the identified reference genes. Primers of the two genes were designed according to the aforementioned methods and listed in **Table 1**. To demonstrate the transcriptional regulation of sucrose metabolism, an earlier sampling point at 7 DAA was added and used as control to analyze the expression levels of *CmAIN2* and *CmSPS1*. qRT-PCR reactions were performed according to the aforementioned methods. The 2^-ΔΔCt^ method was used to calculate the relative expression level. The best reference genes identified by geNorm and NormFinder were used for normalization. Geometric means were calculated for the reference gene combinations and used for normalization. The widely used reference gene *CmCYP7* in melon fruits and the least stable reference gene identified in this study were additionally used for normalization. Three biological and two technical replicates were adopted for the aforementioned measurements at each sampling point.

## Results

### PCR-Amplification Characteristics of the Candidate Reference Genes

A total of 20 genes were selected as the candidate reference genes in this study, which included the six reference genes that previously identified in melon roots, leaves, and stems (Kong et al., 2014b; Sestili et al., 2014). Information on these 20 candidate reference genes is listed in **Table 1**. Primer sequences of the six reference genes previously identified in melon (Kong et al., 2014b; Sestili et al., 2014) were also used in this study. While, primer sequences of the other 14 candidate reference genes were designed.

**FIGURE 1:**
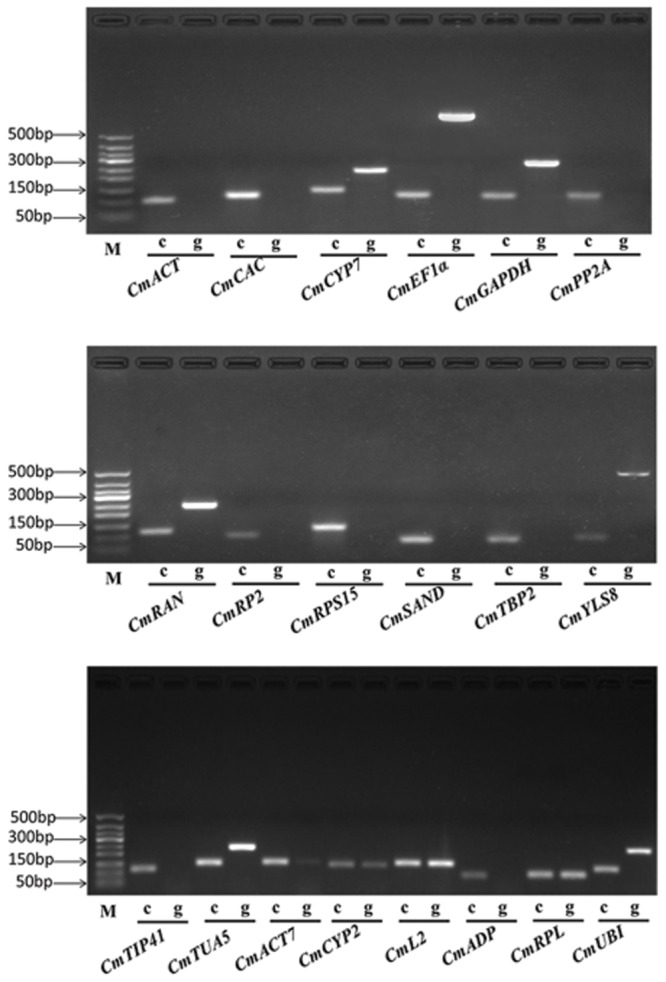
**Polymerase chain reaction (PCR) amplification products of 20 reference genes in 2% agarose gel.** ‘c’ indicates the cDNA template, ‘g’ indicates the gDNA template, ‘M’ indicates the DNA ladder marker.

To improve the specificity of qRT-PCR analysis, one primer of each gene was initially designed to match an exon–exon junction. However, a suitable primer binding site was difficult to find in the exon–exon junction regions of some genes. Primers of the said genes were then located on the exons separated by an intron. Amplification specificity of the 20 genes was determined by a 2% agarose-gel electrophoresis with cDNA and genomic DNA (gDNA) as templates, respectively (**Figure 1**). The results showed that only the expected products were amplified with cDNA templates and no products were amplified with gDNA templates for *CmACT, CmCAC, CmPP2A, CmRP2, CmRPS15, CmSAND, CmTBP2*, and *CmTIP41*. Meanwhile, *CmCYP7, CmEF1α, CmGAPDH, CmRAN, CmYLS8*, and *CmTUA5* amplified specific products from both cDNA and gDNA templates. However, the amplicons from gDNA templates containing an intron are larger than those from cDNA templates, demonstrating the success of primer design and the absence of gDNA contaminations in the cDNA samples. Among the six previously identified reference genes in melon, *CmACT7, CmCYP2, CmL2*, and *CmRPL* amplified the same products on both gDNA and cDNA templates, whereas, *CmADP* had no products and *CmUBI* generated a larger product on gDNA templates, respectively. Meanwhile, a single peak in the melting curve analysis further supported the specific amplification of each gene. Amplification efficiencies of the 20 genes varied from 93.1% (*CmEF1α*) to 109.3% (*CmL2*). **Table 1** summarizes the primer sequences and amplification characteristics of the 20 genes.

### Expression Stability of the Candidate Reference Genes

**FIGURE 2:**
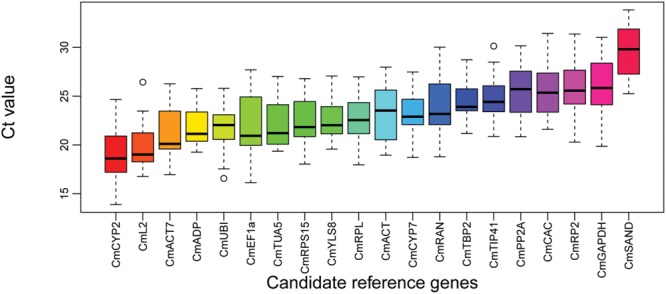
**Boxplot analysis on expression profiles of the candidate reference genes across all 24 samples.** The line across the box represents the median. The boxes represent the 25/75 percentiles. The whiskers show the maximum and minimum values. The circles indicate the outliers.

Expression variations of the 20 genes were examined during the developments of pollinated and CPPU treated fruits and represented as boxplot in **Figure 2**. The mean Ct values for the 20 genes across the 24 samples ranged from 19.0 (*CmCYP2*) to 29.4 (*CmSAND*). *CmADP* exhibited the least expression variation, whereas *CmRP2, CmRAN, CmGAPDH*, and *CmEF1a* showed the highest expression variations. Given the presences of non-biological variations in the experiments, gene expression stability cannot be accurately estimated by direct comparison of the raw Ct values. Therefore, geNorm and NormFinder were used to assess the expression stability.

**FIGURE 3:**
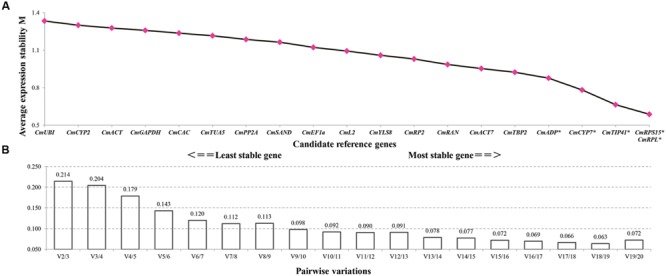
**geNorm analysis of the candidate reference genes.** Genes are ordered by descending M values **(A)**, and the minimum number of reference genes required for reliable normalization is demonstrated by pair-wise variation analysis **(B)**. The asterisks indicate the reference genes needed for more reliable normalization when the cutoff value 0.15 was adopted **(A)**.

GeNorm calculates the expression stability value (M) for each gene and considers that gene with lower M value has higher expression stability. Taking into account the effects of fruit set methods and developmental phases, geNorm ranked *CmRPL* and *CmRPS15* as the pair of best reference genes, and *CmUBI* as the least stable gene (**Figure 3A**). Furthermore, geNorm determines the optimum number of reference genes required for reliable normalization by calculating the pairwise variation values (V). When the pairwise variation (*V_n/n+1_*) is less than 0.15, it is recommended that no additional genes are required for the normalization. Pairwise variation analysis demonstrated that at least five reference genes were required for more reliable normalization, namely, *CmRPL, CmRPS15, CmTIP41, CmCYP7*, and *CmADP* (**Figure 3B**).

**FIGURE 4:**
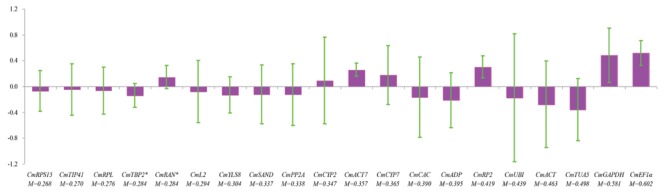
**NormFinder analysis of the candidate reference genes.** The samples were divided into two subgroups according to method of fruit set. The histogram displays the intergroup variation. The error bars represent the intragroup variation. M represents the stability value. Asterisks indicate the combination of two best genes.

NormFinder uses a model-based approach and considers variations across groups to calculate the expression stability for each gene. The gene with lower stability value is top ranked. The samples were divided into fertilized and parthenocarpic groups according to the fruit set methods. The plots of inter- and intragroup variations with respect to fruit set methods for each gene showed that *CmTIP41* had the lowest intergroup variation, whereas *CmACT7* exhibited the lowest intragroup variation (**Figure 4**). *CmRPS15* was determined as the best reference gene with the lowest stability value of 0.268. Meanwhile, NormFinder identified *CmRAN* and *CmTBP2* as the combination of two best reference genes, with minimal combined inter- and intragroup variations. *CmEF1α* was ranked as the least stable reference gene (**Figure 4**).

### Validation of the Identified Reference Genes

**FIGURE 5:**
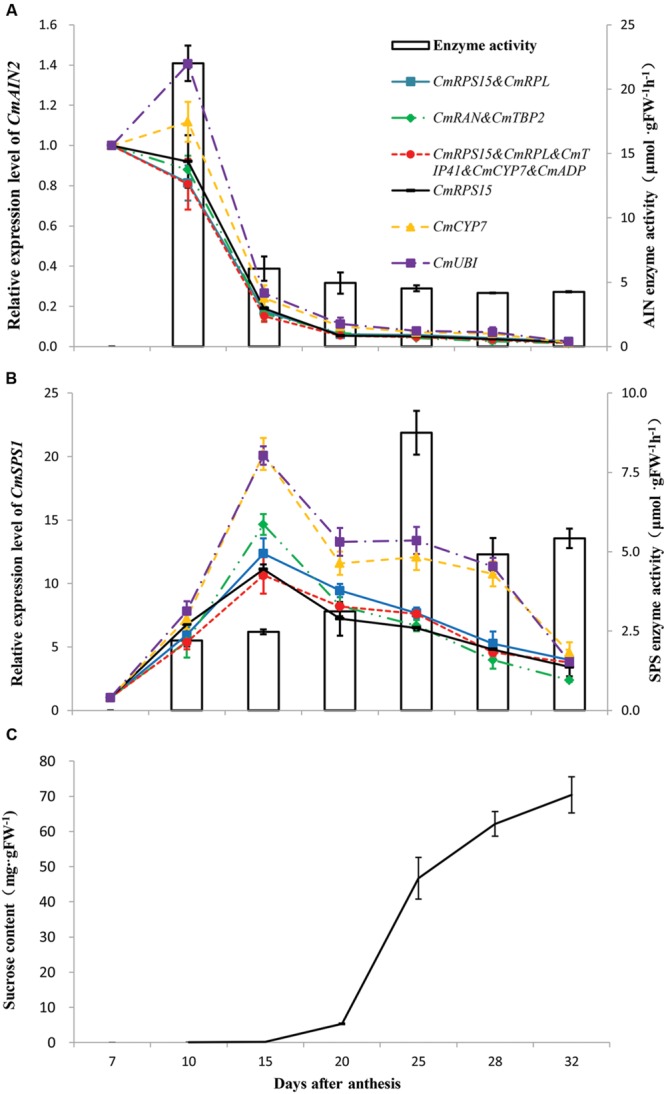
**Validation of the identified reference genes.** AI enzyme activity and *CmAIN2* expression profile **(A)**, SPS enzyme activity and *CmSPS1* expression profile **(B)**, as well as sucrose content **(C)** were measured during melon fruit development. The relative expression levels of *CmAIN2* and *CmSPS1* were normalized by the single best reference gene (*CmRPS15* determined by NormFinder), the pair of best reference genes (*CmRPS15* and *CmRPL* identified by geNorm, and *CmRAN* and *CmTBP2* identified by NormFinder), the multiple reference genes (*CmRPL, CmRPS15, CmTIP41, CmCYP7*, and *CmADP* identified by geNorm), the least stable reference gene (*CmUBI* determined by geNorm), and the previously used reference gene in melon fruits (*CmCYP7*), respectively. Transcript abundance of 7 DAA was used as control. The results are depicted as the mean ± SE (*n* = 6).

Parallel changes of sucrose contents, activities of AI and SPS, and expression patterns of *CmAIN2* and *CmSPS1* were analyzed during fruit ripening (**Figure 5**). The AI activity was highest at 10 DAA and then decreased sharply with fruit ripening (**Figure 5A**). The SPS activity gradually increased from 10 DAA and reached the highest level at 25 DAA, then decreased and kept stable till fruit matured (**Figure 5B**). The sucrose contents were very low before 20 DAA and then gradually increased with fruit ripening (**Figure 5C**). The best genes that determined by geNorm and NormFinder were used to normalize the expression levels of *CmAIN2* and *CmSPS1*, respectively. These genes included the single gene *CmRPS15*, combination of *CmRPS15* and *CmRPL*, combination of *CmRAN* and *CmTBP2*, and multiple genes of *CmRPL, CmRPS15, CmTIP41, CmCYP7*, and *CmADP*. Meanwhile, *CmCYP7* and the least stable gene *CmUBI* determined by geNorm were also used for normalization. The relative expression levels of *CmAIN2* gradually decreased after 7 DAA, and were nearly undetectable after 20 DAA when the best reference genes were used for normalization (**Figure 5A**), which were in agreement with the changing patterns of AI activities. However, when *CmUBI* or *CmCYP7* was used for normalization, the expression levels of *CmAIN2* increased from 7 to 10 DAA and then decreased as fruit ripening. *CmSPS1* was upregulated from 7 to 15 DAA, then gradually downregulated as fruit matured, no matter the stable or less stable genes were used for normalization (**Figure 5B**). However, compared with the expression levels normalized by the best genes, the expression levels of *CmSPS1* were obviously overestimated from 15 to 28 DAA when the less stable gene *CmUBI* or *CmCYP7* was used for normalization.

## Discussion

Expression analysis is a crucial step to gain insight into the biological functions of genes. However, systematic validation of reference genes has not been performed for qRT-PCR-based gene expression studies in melon fruits to date. The use of non-validated reference genes for normalization does not meet the prerequisites of qRT-PCR and thus may introduces bias into the final results (Gutierrez et al., 2008; Bustin et al., 2009). In this study, 20 candidate reference genes were systematically examined for their potential use as reference genes in melon fruits.

To improve the specificity of qRT-PCR analysis, gene-specific primers locating at the exon–exon junctions or two adjoining exons were designed for the 14 candidate reference genes in this study. The similar strategy was also used in *Arabidopsis* (Czechowski et al., 2005), strawberry (Amil-Ruiz et al., 2013), and watermelon (Kong et al., 2014a, 2015). The primer pairs developed in the present study can detect or control the potential gDNA contaminations in cDNA samples, and thus can be used in the related studies.

NormFinder and geNorm are the widely used algorithms to identify suitable reference genes. Both geNorm and NormFinder demonstrated that the previously used *ACT, CYP7*, and *GAPDH* were not the optimal reference genes in melon fruits. *CmACT* and *CmGAPDH* were ranked the second to fourth from the last by geNorm and NormFinder. Only *CmCYP7* was in the multiple reference genes determined by geNorm. Moreover, the unstable expression of these genes has been demonstrated in the fruits of other crops, such as papaya (Zhu et al., 2012) and blueberry (Die and Rowland, 2013). As far as the six best reference genes that identified in the melon roots, leaves, and stems are concerned (Kong et al., 2014b; Sestili et al., 2014), only *CmRPL* was in the pair of best genes and *CmADP* was in the multiple genes that determined by geNorm in melon fruits, indicating the organ-specific characteristics of reference genes and importance of identifying appropriate reference genes specifically for melon fruits.

NormFinder identified *CmRPS15* as the single best reference gene, and geNorm identified *CmRPS15* and *CmRPL* as the pair of best reference genes in melon fruits. *RPS15*, initially called as *rig*, has been proven to be a housekeeping gene in human (Shiga et al., 1990; Kitagawa et al., 1991) and was identified as the suitable reference gene in many cases in mammals (Bionaz and Loor, 2007; Kumar et al., 2012). The housekeeping feature of *PRS15* also supported the suitability of *CmRPS15* as reference gene in melon fruits. Meanwhile, *RPS2*, the other member of *RPS* gene family was also identified as the suitable reference gene in banana fruits (Chen et al., 2011). The ribosomal protein related genes *CmPRL* and *CmL2* were ranked among the suitable reference genes in melon roots, stems, and leaves in the previous studies (Kong et al., 2014b; Sestili et al., 2014). In this study, *CmRPL* was also identified as one of the suitable reference genes in melon fruits, suggesting that ribosomal protein related genes were potentially the widely applicable reference genes in melon. NormFinder identified *CmTBP2* and *CmRAN* as the combination of two best reference genes, Similarly, *TBP2* was ranked among the suitable reference genes in papaya fruits (Zhu et al., 2012). However, *RAN* was not reported as the suitable reference genes in the fruits of other crops in the previous studies, indicating the importance of identifying suitable reference genes for specific species.

Acid invertase and SPS were considered the determinants of sucrose concentration in developing melon fruits (Hubbard et al., 1989; Burger and Schaffer, 2007). Thus, parallel analyses of the activities of AI and SPS, and expression patterns of their respective encoding genes, as well as sucrose accumulation during melon fruit ripening, can provide reliable measurement to test the suitability of the identified reference genes. Increased sucrose contents accompanied by the decreased AI activities were observed as fruit matured. Meanwhile, SPS activities also tended to be increased during fruit ripening, although its highest level occurred at 25 DAA. These results were in general agreement with the previous reports on sucrose metabolism in melon fruits (Hubbard et al., 1989; Burger and Schaffer, 2007; Dai et al., 2011). The expression patterns of *CmAIN2* were nearly identical regardless the best single, pair of, or multiple reference genes were used for normalization. They exhibited positive correlation with the developmental changes of AI activity and negative correlation with the sucrose accumulation during fruit development. Similar results were also observed in ripening melon fruits by deep sequencing analysis (Dai et al., 2011). No obvious correlation was observed between expression patterns of *CmSPS1* and SPS activities or sucrose contents during fruit development regardless the stable or unstable reference genes were used for normalization, suggesting that *CmSPS1* was possibly not the major gene regulating sucrose metabolism on transcriptional level. *CmUBI* was ranked as the least stable gene by geNorm. Although *CmCYP7* was among the multiple reference genes determined by the pairwise variation analysis of geNorm, its expression stability was less than that of the identified best single or pairs of genes. Compared with the best single reference gene or gene combinations, the expression levels of *CmAIN2* and *CmSPS1* were overestimated at some sampling points when the less stable gene *CmUBI* or *CmCYP7* was used for normalization, which highlighted the importance of selecting the systematically validated reference genes in qRT-PCR analysis. Although different best single reference gene or gene combinations were identified by geNorm and NormFinder, the expression patterns of *CmAIN2* or *CmSPS1* were nearly identical when these identified genes were used for normalization, and in general agreement with the previous reports or the changing patterns of their respective encoding protein activities, demonstrating the suitability of the identified reference genes in melon fruits.

## Conclusion

The suitability of 20 genes for their potential use as reference genes in melon fruits was assessed in this study. For more reliable normalization, the multiple genes including *CmRPL, CmRPS15, CmTIP41, CmCYP7*, and *CmADP* were required in melon fruits. However, taking account the cost and similar normalization results as the multiple reference genes, *CmRPS15* alone or together with *CmRPL*, or combination of *CmRAN* and *CmTBP2* were the preferred reference gene or reference gene combinations that can be used to replace the non-validated reference genes that previously used in melon fruits. This study offers a reference gene selection guideline specifically for melon fruits and provides valuable information for further studies on the transcriptional regulation of sucrose metabolism during melon fruit ripening.

## Author Contributions

QK and ZB conceived and designed the experiments. LG, LC, YL, and HS performed the experiments. QK and LG analyzed the data. YH contributed reagents/materials. QK wrote the manuscript.

## Conflict of Interest Statement

The authors declare that the research was conducted in the absence of any commercial or financial relationships that could be construed as a potential conflict of interest.

## Footnotes

1 https://melonomics.net/

2 http://primer3plus.com/cgi-bin/dev/primer3plus.cgi

3 http://www.r-project.org/
